# China’s Air Quality and Respiratory Disease Mortality Based on the Spatial Panel Model

**DOI:** 10.3390/ijerph14091081

**Published:** 2017-09-18

**Authors:** Qilong Cao, Ying Liang, Xueting Niu

**Affiliations:** 1 Business School, Changzhou University, Changzhou 213164, China; cql1086@cczu.edu.cn; 2 Department of Social Work and Social Policy, School of Social and Behavioral Sciences, Nanjing University, 163 Xianlin Avenue, Qixia District, Nanjing 210023, China; 3 Department of Sociology, School of Social and Behavioral Sciences, Nanjing University, 163 Xianlin Avenue, Qixia District, Nanjing 210023, China; mg1607015@smail.nju.edu.cn

**Keywords:** air quality, PM_2.5_, spatial data, mortality, China

## Abstract

*Background*: Air pollution has become an important factor restricting China’s economic development and has subsequently brought a series of social problems, including the impact of air pollution on the health of residents, which is a topical issue in China. *Methods*: Taking into account this spatial imbalance, the paper is based on the spatial panel data model PM_2.5_. Respiratory disease mortality in 31 Chinese provinces from 2004 to 2008 is taken as the main variable to study the spatial effect and impact of air quality and respiratory disease mortality on a large scale. *Results*: It was found that there is a spatial correlation between the mortality of respiratory diseases in Chinese provinces. The spatial correlation can be explained by the spatial effect of PM_2.5_ pollutions in the control of other variables. *Conclusions*: Compared with the traditional non-spatial model, the spatial model is better for describing the spatial relationship between variables, ensuring the conclusions are scientific and can measure the spatial effect between variables.

## 1. Introduction

Clean air is a basic demand of human health and well-being [1], yet air pollution is still a serious problem globally, especially in fast-developing countries such as China. According to China’s environmental data released by the Ministry of Environmental Protection of China in 2015, the primary air pollutants in 339 cities with air pollution monitoring included PM_2.5_, O_3_, and PM_10_ [2]. The emergence of air pollution is affected by a variety of factors, such as coal or straw combustion, traffic emissions [3], and long-distance transportation of dust [4].

Air pollution has a serious impact on people’s physical and mental health. Long-term or short-term survival in the context of excessive PM_2.5_ will increase the risk of people suffering from various diseases, such as diabetes, increased hypertension [5], cardiovascular disease [6], cardiopulmonary disease [7] and lung cancer [8,9], all resulting in an increase of mortality [10]. Air pollution can also affect the weight of newborn babies [11]. If pregnant women are excessively exposed to PM_2.5_ in the air, they can experience low birth weight, premature delivery, and other related conditions [12]. Air pollution also adversely affects people’s mental health [13]. Specifically, air pollution can impair neurocognitive functions [14] and lead to people suffering from depression more easily [15]. Giovanis and Ozdamar’s study shows that air pollution has a negative effect on people’s life satisfaction [16] and reduces their subjective well-being [17].

Air pollution contributes to premature death globally. The number of deaths due to outdoor air pollution will double by 2050 if no action is taken [18], and serious air pollution also increases the suicide rate [19]. The conclusion has been validated in different meteorological conditions, topographical conditions, and cultural groups [20]. This clearly shows that air pollution causes serious physical and mental health dangers, meaning it requires further research.

These studies use cross-sectional data or panel data for analysis, and initially identified that air pollution will cause some harm to people’s health. However, the use of cross-section data will result in a large number of time-varying characteristics and loss of information which will affect the effectiveness of the sample, so that there is a lack of explanatory research. Although the application of panel data can, to some extent, reflect the characteristics of time evolution of variables, the existence of inter-region air pollution leads to the spatial correlation among variables. Therefore, the simple use of panel data still has limitations.

### 1.1. The Relations between Air Pollution and Mortality

Scholars have extensively studied the impact of air pollution on people’s health and mortality. Wang et al. took Beijing as a sample to study the effects of air pollution on people’s health five months before and after an Asia-Pacific Economic Cooperation (APEC) meeting in Beijing [21]. Chung et al. used the PM_2.5_ data from the Eastern United States 2000–2006 to explore the relationship between location-specific mortality and the previous year’s PM_2.5_ [10]. Jerrett et al. studied the relationship between mortality and air pollution in the Toronto (Canada) area over the 1992–2002 period [22].

Based on the above, some scholars began to try to use the method of spatial analysis to explore the relationship between air pollution and mortality. Jerrett et al. used spatial analysis to explore the relationship between air pollution and mortality in California and found that PM_2.5_, O_3_, NO_2_, and other pollutants have a positive effect on mortality [23].

Liu et al. explored the relationship between air pollution and mortality in 120 cities in China using spatiotemporal analysis, and found that the air pollution index increased from 2012 to 2013, and the change of mortality from 4% to 7% can be explained by the air pollution index [24]. All of the above studies use the air pollution index (API) as the standard to judge the air quality, and collectively measure the combined effects of PM_2.5_, O_3_, NO_2_, SO_2_, and other pollutants on mortality. Compared with the existing multiple indicators or the comprehensive indicators of the air pollution index as the proxy variable of air pollution, PM_2.5_ was selected as the key variable of air pollution for this paper. The main reasons are as follows: (1) the proportion of PM_2.5_ pollution in China’s air pollution sources is increasingly becoming an important pollution source instead of sulphide and nitrogen oxides, and it has been given greater attention by the Chinese [25]; (2) the main focus of this study is the relationship between mortality of respiratory diseases and air pollution. Compared with air pollutants such as sulphide and nitrogen oxides, PM_2.5_ respirable particulate contamination has a greater impact on inducing respiratory disease [26]. For this reason, the PM_2.5_ index is used to measure the relationship between this respirable particulate contamination and mortality of respiratory disease more precisely (usually in aerodynamics, particles below 10 microns in diameter are called PM_10_, also known as respirable particulate matter; particles below 2.5 microns in diameter are called PM_2.5_, also known as particles into the lungs.).

### 1.2. Research Purpose and Hypothesis

The main aim of this paper is to study the spatial effect and the relationship between air quality and mortality of respiratory disease in 31 provinces of China from 2004 to 2008. The main contributions of this paper are as follows: (1) spatial econometrics methods are used. The spatial effect of air quality and mortality of respiratory disease and their correlation were investigated using three different spatial econometric models. Compared with the traditional non-spatial model, the spatial model could describe the spatial relationship between variables better, ensuring the conclusions of the study were more scientific; (2) in this paper, spatial panel data are used for the study. The paper is based on the data of 31 provinces in China. The time span is from 2004 to 2008, and the results of large scale space-time are more persuasive.

As a developing country with rapid economic development, the problem of air pollution in China has sparked the concerns of researchers. The relevant research reveals the temporal and spatial effects and characteristics of air pollution from different aspects and perspectives. The majority of the existing research on China’s air pollution has the view that China’s air pollution presents a certain degree of spatial clustering. China’s air pollution in the eastern region was significantly higher than the western one, showing the agglomeration characteristics of pollution in the eastern region [27,28].

Compared with the western region, China’s central and eastern regions, especially the eastern one, are more developed. China’s major economic circles, such as the Beijing-Tianjin-Hebei economic circle, the Yangtze River Delta economic circle, and the Pearl River Delta economic circle are located in China’s eastern regions. China’s policies lack long-term environmental planning and competition among different provinces for economic growth, and are often at the expense of environmental degradation.

In addition, urbanisation has been strongly advocated by the Chinese government. Rapid urbanisation has led to a rapid growth in urban vehicle ownership, a large number of enterprises gathering around the city, motor vehicle exhaust emissions and industrial emissions, increasing the air pollution in economically developed areas such as Beijing, Shanghai and other eastern districts [28]. Therefore, the hypotheses of this paper are as follows:

**Hypothesis 1**: *PM_2.5_ pollution in China presents a certain characteristic of agglomeration and spatial autocorrelation in geographical space, and it is the main haze pollution gathering place in China’s eastern region*.

**Hypothesis 2**: *China’s respiratory disease mortality has a similar regional distribution as PM_2.5_ pollution, and in addition, a positive correlation between the space is presented between PM_2.5_ concentrations and the mortality of respiratory disease in China*.

## 2. Materials and Methods

### 2.1. Variables and Data Source

In this paper, the dependent variable is the mortality of respiratory diseases and the independent variable is air pollution, which is indicated by PM_2.5_. Studies have shown that there are other factors that can affect the mortality rate of respiratory diseases, such as the level of economic development of a region [29], per capita health care costs [30], population density [31], and the level of medical care [32]. Taking into account the impact of these factors, this paper selects the per capita GDP of each province, the per capita medical expenses of each province, the population density of each province and the number of general hospitals in each province as the control variables.

In order to avoid the heteroscedasticity, this paper considers the natural logarithm of large variables. We log-transformed the per capital GDP of each province (China Statistics Yearbook, 2005–2009), which was labelled as lngdp; medical expenses of each province (China Health Statistics Yearbook, 2005–2009), which was labelled as lncost; number of general hospitals in each province (China Health Statistics Yearbook, 2005–2009), which was labelled as lnhos. In addition, we defined the population density (China Statistics Yearbook, 2005–2009) as the ratio of person counts to square kilometres, and labelled it as popudens.

### 2.2. Data of PM_2.5_

As there is a serious lack of data on Chinese PM_2.5_ levels, this paper refers to international data for the study. Previously, Van Donkelaar et al. used satellite data to produce the world’s first PM_2.5_. concentration map to show high haze areas in East Asia and North Africa, including northern, eastern and central China [33]. The Bartel Institute and the International Geoscience Information Network Centre at Columbia University used the satellite-mounted device to measure aerosol optical thickness (AOD) with the help of Donkelaar et al. [34] (Source, International Geoscience Information Network Center, Columbia University (2004–2008), http://sedac.ciesin.columbia.edu/data/sets/browse).

This paper downloaded the global PM_2.5_ map of raster data format from the website, and then used the ArcGis10.2 software (Environmental Systems Research Institute, Redlands, CA, USA) to process the PM_2.5_ data of 31 administrative regions in China, taking 0.1° × 0.1° as the sampling point, PM_2.5_ values of each grid are extracted.

### 2.3. Mortality of Respiratory Disease

The explanatory variable in this paper is mortality of respiratory disease, which is derived from the China Disease Detection System Database of Causes of Death Monitoring Report. The DSP (disease surveillance point) system currently includes 161 monitoring points, covering 70 million people. In this paper, the mortality of respiratory diseases per 10,000 inhabitants in China was selected as the explanatory variable from the “China Disease Detection System Death Monitoring Network Report Database” for 2004–2008, named “respdeath” to study the relationship between PM_2.5_ and mortality of respiratory disease (Data Sources, National Monitoring System for Disease Surveillance Database (2004–2008) http://www.phsciencedata.cn/Share/index.jsp). In addition, as with the description of the China Disease Detection System Database, the present study uses the numbers of respiratory disease deaths per ten thousand people to identify clusters.

### 2.4. Analysis Model

The concept of space econometrics was first put forward by Paelinck, after the efforts of scholars such as Anselin to develop and gradually form a framework for a space econometrics system. The first step of spatial analysis is called spatial exploratory analysis, and its purpose is to determine whether the research objectives are spatially interdependent using space exploration tools. If spatial interdependence exists, spatial measurement methods can be sequentially used to study the spatial effect of variables. On the contrary, the traditional measurement method can be used. The analysis of the dependence of spatial variables mainly tests whether spatial autocorrelation exists among the variables, including the global spatial correlation test and the local spatial correlation test. Moran’s *I* index is the main statistical tool of global spatial correlation testing. Moran’s *I* scatter plot is the main tool of the local spatial correlation test [35]. The Moran index is calculated as follows:

(1) $$I=\frac{\sum_{i=1}^{n}\sum_{j=1}^{n}w_{ij}\left(X_{i}-\bar{X}\right)\left(X_{j}-\bar{X}\right)}{S^{2}\sum_{i=1}^{n}\sum_{j=1}^{n}w_{ij}}$$

In the above formula, I denotes the global Moran’s *I*, measuring the overall correlation degree of the observed variables in different regions; $S^{2}=\frac{1}{n}\overset{n}{\underset{i=1}{\sum }}{\left(X_{i}-\bar{X}\right)}^{2}$ is the variance of the spatial sample; $\bar{X}=\frac{1}{n}\overset{n}{\underset{i=1}{\sum }}X_{i}$ is the average of the observed variables in different regions; and *w_ij_* is the spatial weight matrix.

Moran’s *I* is used to measure the overall correlation between regions, and values generally range between −1 to 1, and the closer to 1, the higher the degree of spatial positive correlation between regions, that is, similar values in the spatial distribution tend to be concentrated in one region. The closer to −1, the higher the degree of spatial negative correlation between regions. When the exponent is 0 there is no spatial correlation. For the Moran’s *I* index, the critical value of the standard normal distribution can be used for testing. The above test is the global spatial autocorrelation test, however, in order to test the local spatial autocorrelation, the local Moran’s *I* is needed. The main tool is Moran’s *I* scatter plot.

The horizontal axis of the Moran scatterplot is the normalised observation and the vertical axis is the spatial lag of the normalised observations. With the mean as the origin, the Moran scatterplot divides the observation values of each province into four quadrants of agglomeration patterns in order to identify the spatial correlation of a province and its neighbouring provinces, respectively.

In four divisory quadrants, in the first quadrant, the provinces with high levels are clustered by the provinces with the high level (High-High); in the second quadrant, the provinces with low levels are clustered by provinces with high levels (Low-High); the provinces with low levels in the third quadrant are surrounded by low-level provinces (Low-Low); a province with a high level in the fourth quadrant is surrounded by a province with a low level (High-Low).

Thus, for the provinces in the first and third quadrants, the observed variables have similar values and exhibit some spatial positive correlation, whereas in the second and fourth quadrants the observed variables are opposite. If the observed values of the provinces are evenly distributed in the four quadrants, there is no spatial autocorrelation among the observed values of the provinces.

### 2.5. The Setting of the Spatial Weight Matrix and the Spatial Model

#### 2.5.1. The Setting of the Spatial Weight

The prerequisite of spatial econometric analysis is to measure the spatial distance between regions, and the index used is the spatial weight matrix. Note that the spatial data from *n* regions is ${\left\{X_{i}\right\}}_{i=1}^{n}$, and the subscript *i* represents region *i*. The spatial weight matrix *w_ij_* is used to represent the distance between the region *i* and the region *j*. This paper adopts the binary weighting matrix which is widely used in literature and follows the Rook adjacency rule that two regions with the same boundary are regarded as adjacent, otherwise they are regarded as not adjacent. The Rook adjacency rule sets the weight matrix as follows:

If the region *i* is adjacent to the region *j*, the weight matrix is equal to 1;
If the region *i* is not adjacent to the region *j*, the weight matrix is equal to zero;
If the region *i* is equal to the region *j*, the weight matrix is equal to zero.

Since Hainan Province is an island in China, it is not connected with other provinces according to the above definition, so it is prone to error when standardising the spatial weight matrix in the space measurement model. Therefore, this paper defines Guangdong Province, the nearest to Hainan, as the neighbour of Hainan. Therefore, according to the above definition, the weight matrix adjacent information used in this paper is shown in the Table 1.

**Figure 1:**
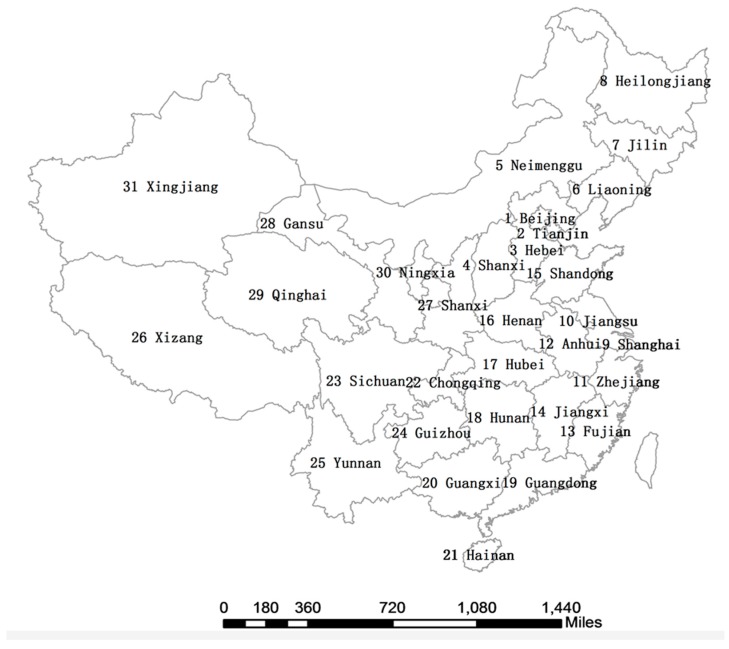
Numbered provinces and cities in China.

Table 1 shows the geographic neighbouring information of 31 provinces and cities in China. The number column is the unique identification code of each region. The region column is the name of each region. The adjacent region code field is the region code adjacent to each region. For example, as number 1 is the Beijing area, the codes of the areas adjacent to Beijing, that is, Tianjin and Hebei Province, are 2 and 3, and so on. According to Table 1, the weight matrix generated according to the Rook rule between provinces in the country can be obtained, that is, the distance between neighbouring provinces is 1, the distance between non-neighbouring provinces is 0, the provinces themselves. The distance is also zero. A map with each province and its corresponding number fitting into Table 1 was shown in Figure 1.

#### 2.5.2. The Setting of the Spatial Model

As a result of the spatial panel data, the following spatial panel data model is established on the basis of considering the spatial effect of the provinces, as well as the individual effect and time effect:

(2) $$\begin{array}{cc} respdeath_{it}= & \alpha +\rho \sum w_{ij}respdeath_{jt}+\delta \sum w_{ij}\sum x_{jt}+\beta_{1}PM2.5_{it}\\ & +\beta_{2}lngdp_{it}+\beta_{3}lnhos_{it}+\beta_{4}lncost_{it}+\beta_{5}popudens_{it}\\ & +\mu_{i}+\gamma_{t}+\varepsilon_{it} \end{array}$$

where *ε_it_ = λ × Σ m_it_ × ε_it_ + ψ_it_*, In the above equation, *α* is the intercept term, *Σ w_ij_ × respdeath_jt_* represents the spill-over effect of space, its *ρ* measures the size of the spatial effect, *δ × Σ w_ij_ × Σ x_jt_* indicates the spatial lag of the explanatory variables, *Σ x_jt_* is the explanatory variable involved in this paper, namely the air quality index *PM*_2.5*it*_, the per capita income indicator is *lngdp_it_*, the per capita medical expenses is *lncost_it_*, the number of general hospitals in each province is *lnhos_it_*, and the population density index is *popudens_it_*, *μ_i_* denotes the random disturbance term of the individual effect, *γ_t_* denotes the random disturbance term of the time effect, *ε_it_* denotes the spatial error term, and *m_it_* denotes the spatial weight matrix of the error term.

The above is a general model of the spatial panel data, according to spatial econometrics, spatial data dependencies can be divided into three types: Spatial Lag Model (SLM), Spatial Error Model (SEM) and Spatial Durbin Model (SDM). For the general model established in this paper, according to the relevant parameter settings it can be divided into the following three models:

(1) If λ = 0, then the model is called the Spatial Durbin Model (SDM). This model assumes that the explained variable of the region *i* depends on the explained and explanatory variables of its neighbouring regions and does not consider the spatial relationship between the error terms. In this model, the coefficient *ρ* examines the influence of the spatial lag of the explained variables on the explained variables, and the coefficient *δ* examines the spatial effects of the spatial lag of the explanatory variables on the explained variables.

(2) If λ = 0 and *δ* = 0, then the model is called the Spatial Lag Model (SLM). The model assumes that the explained variables of region *i* are dependent on the explanatory variables of their neighbouring regions, regardless of the spatial relationship between explanatory variables and error terms of adjacent regions. In this model, the coefficient *ρ* examines the effect of the spatial lag of the explained variable on the explained variable.

(3) If *ρ* = 0 and *δ* = 0, then the model is called the Spatial Error Model (SEM). This model considers that there exists spatial dependency between the disturbance terms, which are not included in explanatory variables, however, they have spatial correlations on the missing variables that affect the explanatory variables. The model assumes that the explained variables of region *i* may be affected by unobservable random shocks. In this model, the coefficient λ examines the influence of the spatial lag of the disturbance term on the explained variables.

Due to the spatial effect, if the three models are still estimated by OLS, the estimation of the coefficients will be partial or invalid. In order to obtain robust results, in this paper the maximum likelihood estimation method is used to estimate the above models separately according to [36].

## 3. Results

### 3.1. Descriptive Statistics

Table 2 shows the results of descriptive statistics for the variables used in this paper. The variable respiratory mortality rates the death toll of respiratory diseases per 10,000 people, with an average of 0.61 per 10,000 persons, a maximum of 3.72 per 10,000 persons and a minimum of 0.01 per 10,000.

PM_2.5_ represents the concentration of PM_2.5_ in the air, the unit is μg/m^3^. According to the data in the table, the average concentration of PM_2.5_ in China in 2004–2008 was 40.67 μg/m^3^, the maximum value was 85.40 μg/m^3^, and the minimum value was 4.14 μg/m^3^. Other descriptive statistics for the control variables are also shown in Table 3.

### 3.2. Spatial Distribution Maps

**Figure 2:**
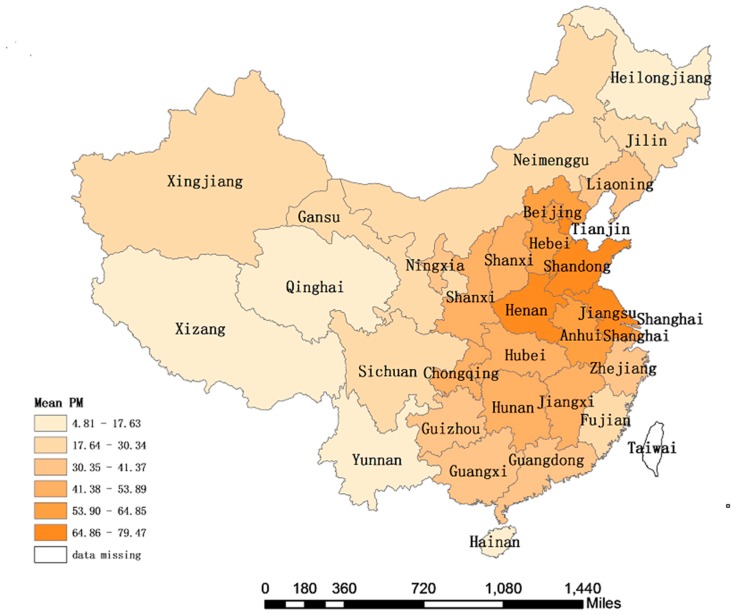
Geographical distribution of average PM_2.5_ in various provinces of China from 2004 to 2008.

Figure 2 shows the geographical distribution of PM_2.5_ concentrations in China from 2004 to 2008, with different colour depths representing different concentrations of PM_2.5_. The darker the colour, the greater the concentration of PM_2.5_. In Figure 2, PM_2.5_ concentrations in 31 Chinese provinces and municipalities are divided into six grades, with the first grade being 64.86–79.47 μg/m^3^, which is the region with the most severe PM_2.5_ concentration as the darkest areas of colour.

It can be seen from Figure 2 that PM_2.5_ pollution is most serious in several provinces in eastern and central China. The second grade is 53.90–64.85 μg/m^3^, and has many polluted areas. As can be seen in Figure 1, these regions are mainly Beijing, Shanghai and Hebei provinces. The concentration value is 41.38–53.89 μg/m^3^ in the third level, as can be seen in Figure 1, and the provinces in this category are mainly in the central provinces of China. The concentration value is 30.35–41.37 μg/m^3^ in the fourth level. It is more geographically dispersed, in addition to some provinces in southern China, including the northeast of Liaoning Province. The concentration value is 17.64–30.34 μg/m^3^ in the fifth level. In addition to the provinces of north-western China, it also includes Jilin Province in the north east of China and Fujian Province in southern China. The concentration value is 4.81–17.63 μg/m^3^ in the sixth level. It is more geographically dispersed, mainly in remote areas of China, such as Hainan Province, Heilongjiang Province, Qinghai Province and Tibet, etc.

**Figure 3:**
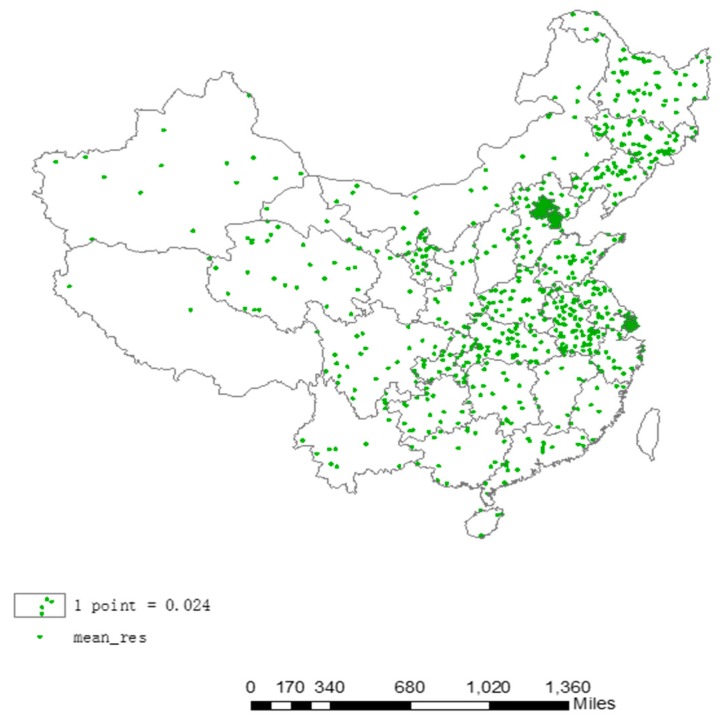
Dot density map of the average respiratory disease mortality for the 2004–2008 Chinese provinces.

Figure 3 is the dot density map of the average respiratory disease mortality rate for each province in China from 2004 to 2008 (with Taiwan data missing). Each point represents 0.02 units, that is, each point represents 0.02 people per ten thousand deaths due to respiratory diseases, the greater the density of points, the higher the respiratory disease mortality in the region, and the opposite applies for the lower.

As can be seen in Figure 3, in 2004–2008, the regions with the highest incidences of respiratory disease mortality in China are the eastern and central regions, whereas respiratory disease mortality in the western regions is relatively low. Specifically, Beijing, Tianjin and Shanghai have the highest respiratory disease mortality and the highest density point. Among them, the average mortality rate of respiratory diseases in Beijing from 2004 to 2008 was 2.25 per 10,000 people, and in Tianjin from 2004 to 2008, the average mortality rate of respiratory diseases was 1.18 per 10,000 people, while the average mortality rate of respiratory diseases was 2.86 per 10,000 people in Shanghai from 2004 to 2008. Other areas (apart from the central provinces of China) with relatively high respiratory disease mortality were 1.19 per 10,000 people in Anhui Province, 1.38 per 10,000 people in Hubei Province, and it includes the three north-eastern provinces of China, that are 1.27 per 10,000 people in Jilin Province, 1.10 per 10,000 people in Heilongjiang Province, 0.91 per 10,000 people in Liaoning Province. Relatively speaking, respiratory disease mortality of the western provinces was much lower, for example for Tibet it was only 0.05 per 10,000 people, and in the Gansu Province 0.17 per 10,000 people, a relatively low level. Table 3 fitting these descriptions is as follows:

### 3.3. Space Exploratory Analysis

#### 3.3.1. Mortality of Respiratory Diseases in China and the Global Spatial Autocorrelation Test of PM_2.5_ Based on Moran’s *I* Index

The following table shows the statistical data of Moran’s *I* for annual respiratory disease mortality and PM_2.5_ in China from 2004 to 2008. According to Table 4, it can be seen that the Moran’s *I* of China’s respiratory disease mortality was 0.210 in 2004, 0.204 in 2005, 0.201 in 2006, and 0.187 in 2007 respectively. Moran’s *I* of 2004–2007 was significant at the 5% level, while Moran’s *I* in 2008 was 0.152 and the *p* value was 0.060, which was significant at the level of 10%.

The statistical index of PM_2.5_ spatial clustering in 2004–2008 can be seen from the above table, that is, the Moran’s *I* is above 0.5, and all are significant at the level of 1%. Therefore, China’s PM_2.5_ also shows a positive spatial clustering. The results in Table 4 also correspond to the geographic distribution of PM_2.5_ for the provinces of 2004–2008 in Figure 1 and the dot density map of the average respiratory disease mortality for the provinces in China from 2004 to 2008. The results in Table 4 indicated that, globally, mortality of respiratory disease is correlated in the space between regions, as well as PM_2.5_.

#### 3.3.2. Chinese Respiratory Disease Mortality and Local Spatial Autocorrelation Test of PM_2.5_ Based on Moran’s *I* Scatter Plot

**Figure 4:**
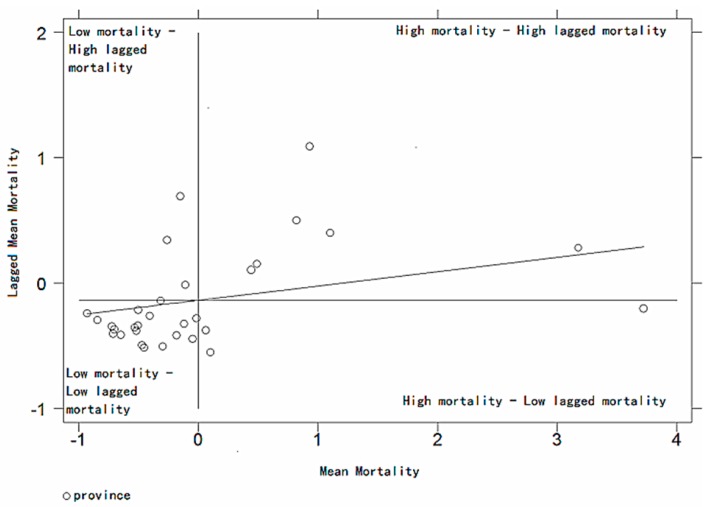
Moran scatter plots for average respiratory disease mortality in 2004–2008.

Figure 4 is the Moran scatter plot of the average respiratory disease mortality from 2004 to 2008 in China. As can be seen from Figure 4 that there are six provinces in the first quadrant where the average respiratory disease mortality from 2004 to 2008 in China is distributed, and 21 provinces in the third quadrant, three provinces in the second quadrant, and only one province in the fourth quadrant. This shows that the average mortality of respiratory disease from 2004 to 2008 in China mainly presents the phenomenon of High-High and Low-Low aggregation, only minor provinces present the phenomenon of High-Low and Low-High aggregation. Overall, the Moran scatter plot of average mortality of respiratory disease in China from 2004 to 2008 shows that China’s respiratory disease mortality shows a certain degree of local spatial autocorrelation characteristics.

**Figure 5:**
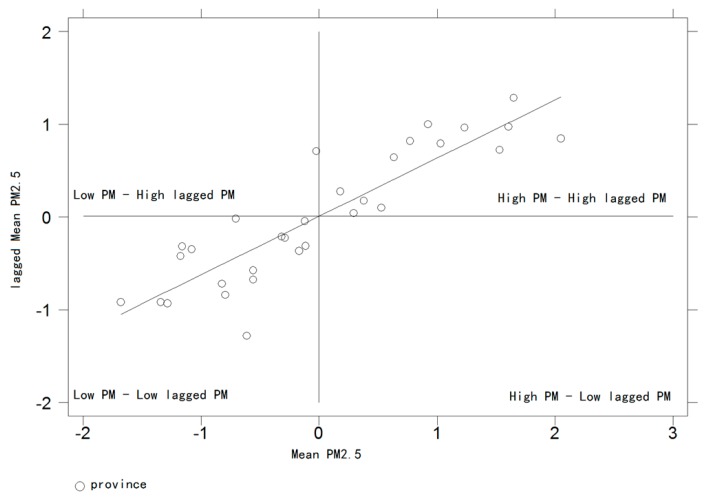
Moran scatterplot for average PM_2.5_ in 2004–2008.

Figure 5 is the Moran scatter plot of average PM_2.5_ in China from 2004 to 2008, and it can be seen from Figure 4 that the Chinese average PM_2.5_ from 2004 to 2008 is mainly distributed in the first and third quadrants, with 15 provinces in the first quadrant, 17 provinces in the third quadrant, but no observed values of provinces are distributed in the second and fourth quadrants. It shows that the average PM_2.5_ from 2004 to 2008 in China presents a very strict phenomenon of High-High and Low-Low aggregation and it also exhibits a very stable relationship between the spatial agglomeration.

### 3.4. The Empirical Results

Next, we performed the spatial effect analysis. In Table 5, the coefficient *ρ* is the spatial effect estimated by the Spatial Durbin Model and the Spatial Lag Model. The coefficient *λ* is the spatial effect estimated by the Spatial Error Model. It can be seen from Table 5 that the spatial effect sizes estimated by these three models are 0.5027 for *ρ* (*p* < 0.01) in SDM, 0.5078 for *ρ* (*p* < 0.01) in SLM, and 0.5912 for *λ* (*p* < 0.01) in SEM, respectively, and they all pass the 1% level of significance test, showing a very strict positive spatial correlation. Thus, all of these three models indicate that the spatial effect exists significantly. Specifically, the Spatial Durbin Model and the Spatial Lag Model indicates the spatial effects of the spatial lag of the explained variables on the explained variables, which can be defined as the spatial autocorrelation effect. Spatial Error Model indicates the spatial effects of the spatial lag of the disturbance term on the explained variables, which can be defined as Spatial Error Dependence Effect [36,37].

It can be concluded that explanatory variables such as air quality and per capita income were related to the mortality of respiratory disease significantly in all of the three models, population density was related to the mortality of respiratory disease significantly in SDM and SEM, while per capita medical expenses and the number of general hospitals in adjacent areas do not have a significant impact on the mortality of respiratory disease in the region. In SLM, the spatial effect of the coefficient *ρ* is 0.5078, which is significant at the 1% level. In SEM, the coefficient *λ* is 0.5912 and is significant at the level of 1%, and it is considered that the spatial lag of the unobservable random shock terms has a certain influence on the mortality of respiratory disease in this region.

In summary, the estimates of these models show that China’s respiratory disease mortality rate has a significantly positive spatial correlation, that is, areas with higher mortality rates are clustered together, and areas with lower mortality rates are clustered together. The reasons for the concentration of mortality of respiratory diseases in China are discussed next by analysing the significance of explanatory variables.

Thirdly, the interpretation of the coefficient estimates. In SDM and SLM, the estimated values of the explanatory variables PM_2.5_ are 0.0281 and 0.0289 respectively, which are all significant at the level of 1%. In SEM, the estimated value of the explanatory variable PM_2.5_ is 0.0205, which is significant at the level of 5%. The results of these three models all indicate that PM_2.5_ has a significantly positive effect on the mortality of respiratory diseases in China. It can also explain the reason for spatial correlation of mortality of respiratory diseases in China, that is, PM_2.5_ can be regarded as one of the important factors inducing respiratory diseases.

## 4. Conclusions

From Figure 2 showing the geographical distribution of PM_2.5_ for 2004–2008 in China, and Figure 5 showing the Moran scatterplot of average PM_2.5_ for 2004–2008, it can be seen that PM_2.5_ in China has an obvious spatial agglomeration feature, leading to a higher respiratory disease mortality in agglomeration areas, and the accumulation of respiratory disease mortality in the region. In addition, the explanatory variable lngdp shows a 1% significance under the SDM, and a 5% significance under SLM and SEM. It indicates that regions with higher per capita income levels also have higher respiratory disease mortality.

The explanation for this result can be found in Figure 2. Most of the regions with high per capita income in China are in the eastern part of China, such as the capital economic circle composed of Beijing and Tianjin, and the Yangtze River Delta region composed of Jiangsu, Shanghai and Anhui. These areas are also areas with higher concentrations of PM_2.5_ in China, so there is a positive correlation between areas with higher per capita income and higher respiratory mortality. Finally, from the significance level, the impact of per capita medical expenditure and the number of general hospitals on respiratory diseases in China is not significant, population density is only significant in the Spatial Doppler model, and not significant in other models.

As a country with a serious air pollution problem, Chinese residents are exposed to the problems air pollution causes. The health problems suffered by residents are becoming more and more concerning, as noted by the government, people and scholars. Based on the spatial panel data model, the relationship between PM_2.5_ and mortality of respiratory disease in China was studied using data from 31 provinces in China from 2004 to 2008. The conclusions of this paper can be summarised in two aspects:

(1) Considering geographical distribution, PM_2.5_ in China is mainly distributed in the eastern part of the country, as well as part of the central region. Further spatial analysis shows that China’s PM_2.5_ not only has a global spatial correlation but also has a local spatial correlation, showing a significant spatial clustering, confirming hypothesis 1. As mentioned above, the central and eastern regions of China are more economically developed, with a large amount of economic resources, however, they also have a large number of pollution sources, automobile exhausts, industrial waste gas, etc., which have caused pollution in China’s central and eastern regions.

(2) The geographical distribution of respiratory disease mortality in China and the geographical distribution of China PM_2.5_ have a certain similarity, that is, higher concentrations of PM_2.5_, and respiratory disease mortality appear in the eastern part of China, and areas with lower concentrations of PM_2.5_ and lower mortality of respiratory disease are basically distributed in the western region of China. Further spatial exploratory analysis showed that Chinese mortality of respiratory disease and PM_2.5_ not only have global spatial correlation, but also local spatial correlation.

The global spatial correlation analysis based on Moran’s *I* showed that Chinese respiratory disease mortality shows a spatial positive correlation at the level of 5%, while PM_2.5_ showed a positive spatial correlation at the 1% level. Local spatial analysis based on the Moran scatter plot showed that respiratory mortality and PM_2.5_ in China showed high value and High-High concentration (Low-Low and Low-Low concentration). The results of regression analysis show that the mortality of respiratory diseases in China has a very strict spatial correlation. Thus, Hypothesis 2 is confirmed.

In addition, per capita income is also positively correlated with respiratory morbidity, which can be explained by the geographic distribution of per capita income being consistent with the geographical distribution of PM_2.5_. Finally, from the significance level, the impact of per capita medical expenditure and the number of general hospitals on respiratory diseases in China is not significant, and population density is only significant in the spatial Doppler model, and not significant in other models.

Based on the data analysis for 2004–2008, and a large scale (31 provinces of China), this paper presents the statistical relationship between respiration and air pollution (PM_2.5_) in Chinese residents. PM_2.5_ has a significant positive effect on the mortality of respiratory diseases in China, and PM_2.5_ pollution is one of the most important factors of respiratory diseases for Chinese residents. Compared with the traditional non-spatial model, the spatial model can better describe the spatial relationship between variables, ensuring the conclusions are scientific and can measure the spatial effect between variables.

This paper has found that China’s air pollution and respiratory disease mortality are spatially correlated, with spatial spill-overs, suggesting that air pollution or respiratory disease mortality in a given province is not only related to some variables in that province, but also affected by the neighbouring provinces. The happiness and the quality of life of the people is the first concerns for a country [38,39]. Although the Chinese government has begun to formulate the relevant environmental protection policy, much work is still needed. In the Beijing area, for example, the implementation of traffic travel restrictions has resulted in almost no improvement in air quality. It is suggested that a more reasonable policy should be designed to establish a regional, or even a unified national, environmental restraint system and improve the Chinese resident’s medical facilities, rather than creating simple traffic restrictions in certain areas or shutting down several polluting enterprises.

There are limitations to this study, such as the study time span was limited only to 2008. This limitation matches the mortality data in China, with respiratory mortality data after 2008 being unavailable. The PM_2.5_ data was taken from the University of Columbia International Geoscience Information Network Centre website, and the global PM_2.5_ raster data was extracted from the global PM_2.5_ grid data. It was not taken from China’s domestic monitoring data because China’s large-scale PM_2.5_ test data was not established until 2013, and beforehand the national PM_2.5_ test data is almost completely missing.

## Figures and Tables

**Table 1:** Geographical adjacency information of 31 provinces and cities in China.

Number	Region	Adjacent Region Number	Number	Region	Adjacent Region Number
1	Beijing	2 3	17	Hubei	12 14 16 18 22 27
2	Tianjin	1 3 15	18	Hunan	14 17 19 20 22 24
3	Hebei	1 2 4 5 6 15 16	19	Guangdong	13 14 18 20 21
4	Shanxi	3 5 16 27	20	Guangxi	18 19 24 25
5	Neimenggu	3 4 6 7 8 27 28 30	21	Hainan	19
6	Liaoning	3 5 7	22	Sichuan	17 18 23 24 27
7	Jilin	5 6 8	23	Chongqing	22 24 25 26 27 28 29
8	Heilongjiang	5 7	24	Guizhou	18 20 22 23 25
9	Shanghai	10 11	25	Yunnan	20 23 24 26
10	Jiangsu	9 11 12 15	26	Tibet	23 25 29 31
11	Zhejiang	9 10 12 13 14	27	Shaanxi	4 5 16 17 22 23 28 30
12	Anhui	10 11 14 15 16 17	28	Gansu	5 23 27 29 30 31
13	Fujian	11 14 19	29	Qinghai	23 26 28 31
14	Jiangxi	11 12 13 17 18 19	30	Ningxia	5 27 28
15	Shandong	2 3 10 12 16	31	Xinjiang	26 28 29
16	Henan	3 4 12 15 17 27			

**Table 2:** Descriptive statistics of variables.

Variable	Obs	Mean	Std. Dev.	Min	Max
Respiratory Mortality Rate	155	0.61	0.71	0.01	3.72
PM_2.5_	155	40.67	20.73	4.17	85.40
GDP	155	9.71	0.57	8.37	11.23
Medical Expenses	155	8.42	0.36	7.63	9.55
Hospital Number	155	8.94	0.77	7.19	10.11
Population Density	155	386.97	516.89	2.23	2978.64

Note: Our panel data consist of 31 provinces, autonomous regions and municipalities, and five-year data ranging from 2004 to 2008, therefore, we have 155 observations (31 × 5 = 155).

**Table 3:** Incidence of respiratory disease mortality by region.

Region	Respiratory Disease Mortality (per 10,000 People)	Region	Respiratory Disease Mortality (per 10,000 People)
Beijing	2.25	Jilin	1.27
Tianjin	1.18	Heilongjiang	1.10
Shanghai	2.86	Liaoning	0.91
Anhui	1.19	Tibet	0.05
Hubei	1.38	Gansu	0.17

**Table 4:** Global Moran’s *I* of respiratory disease mortality and PM2.5 for 2004–2008.

Year	Mortality of Respiratory Disease		PM_2.5_	
	Morlan’s *I*	*p*	Morlan’s *I*	*p*
2004	0.210	<0.05	0.577	<0.01
2005	0.204	<0.05	0.558	<0.01
2006	0.211	<0.05	0.571	<0.01
2007	0.187	<0.05	0.576	<0.01
2008	0.152	<0.1	0.559	<0.01

Note: The numbers of respiratory disease death per ten thousand people was used to measure mortality of respiratory disease.

**Table 5:** Spatial model regression results of air quality and respiratory disease mortality.

Parameters to be Evaluated	SDM Estimation Results			SLM Estimation Results			SEM Estimation Results		
	Coef.	Z	*p*	Coef.	Z	*p*	Coef.	Z	*p*
PM_2.5_	0.0281	2.50	<0.01	0.0289	2.54	<0.01	0.0205	2.13	<0.05
GDP	0.6535	2.52	<0.01	0.5497	2.41	<0.05	0.5766	2.03	<0.05
hospital number	−0.1751	−0.66	0.51	−0.2010	−0.62	0.54	−0.2404	−0.96	0.33
medical expensense	−0.5127	−1.36	0.12	−0.6301	−1.28	0.25	−0.6460	−1.09	0.27
population density	0.0043	2.96	<0.01	0.0042	1.47	0.14	0.0042	1.63	0.10
*δ*	−0.0991	−0.68	0.49						
*ρ*	0.5027	6.16	<0.01	0.5078	8.45	<0.01			
*λ*							0.5912	7.82	<0.05
sigma2_e	0.0762	8.62	<0.01	0.0764	3.94	<0.01	0.0773	3.70	<0.05
R^2^	0.45			0.52			0.51		

Note: Coef. represents the estimated coefficient of the arguments. Z represents the z-value which is used to indicate the significance. SDM assumes that the explained variable of the region *i* depends on the explained and explanatory variables of its neighbouring regions; SLM assumes that the explained variables of region *i* are dependent on the explanatory variables of their neighbouring regions; SEM assumes that the explained variables of region *i* may be affected by unobservable random shocks; *δ* indicates the spatial effects of the spatial lag of the explanatory variables on the explained variables; *ρ* indicates the spatial effects of the spatial lag of the explained variables on the explained variables; and *λ* indicates the spatial effects of the spatial lag of the disturbance term on the explained variables.
